# Supplementary material for: New Red-Shifted 4-Styrylcoumarin Derivatives as Potential Fluorescent Labels for Biomolecules
Source: Molecules. 2022 Feb 22;27(5):1461. doi: 10.3390/molecules27051461 (PMC8912076; doi:10.3390/molecules27051461)
Supplement: Supplementary file 1 [file molecules-27-01461-s001.zip › molecules-1599101-SI.pdf]

Supporting Information (SI)

New Red-Shifted 4-Styrylcoumarin Derivatives as Potential Fluorescent Labels for Biomolecules

Raquel Eustáquio[1], João P. Prates Ramalho[2,3], Ana T. Caldeira[1,2], António Pereira[1,2,*]

[1] HERCULES Laboratory and City University of Macau Chair in Sustainable Heritage, University of Évora, Largo Marquês de Marialva 8, 7000-809 Évora, Portugal.
[2] Chemistry Department, School of Sciences and Technology, University of Évora, Rua Romão Ramalho 59, 7000-671 Évora, Portugal.
[3] LAQV-REQUIMTE, University of Évora, Rua Romão Ramalho 59, 7000-671 Évora, Portugal.
* Correspondence: amlp@uevora.pt

Contents:

1 NMR spectra……………….……………………………………..……..…..S2

2 Mass spectra…………………………………………….……………….…S8

3 Quantum chemical calculations…..……………………....…..........……S11

1. NMR spectra

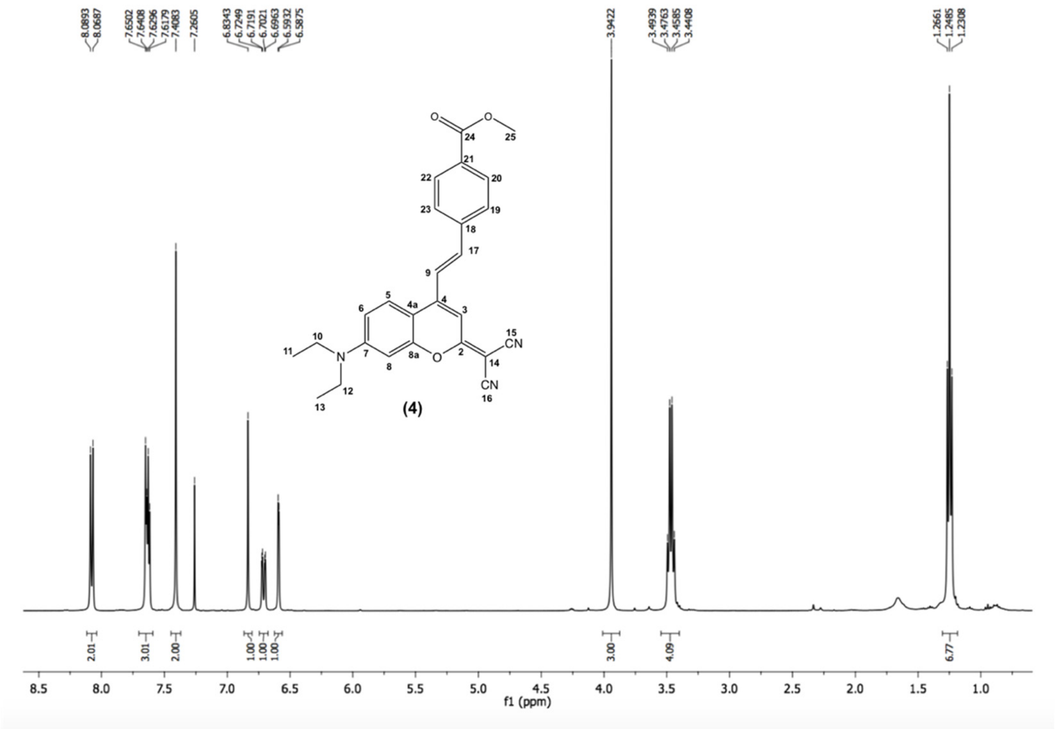

*^{1}H-NMR spectrum of **4** (CDCl₃, 400 MHz)*

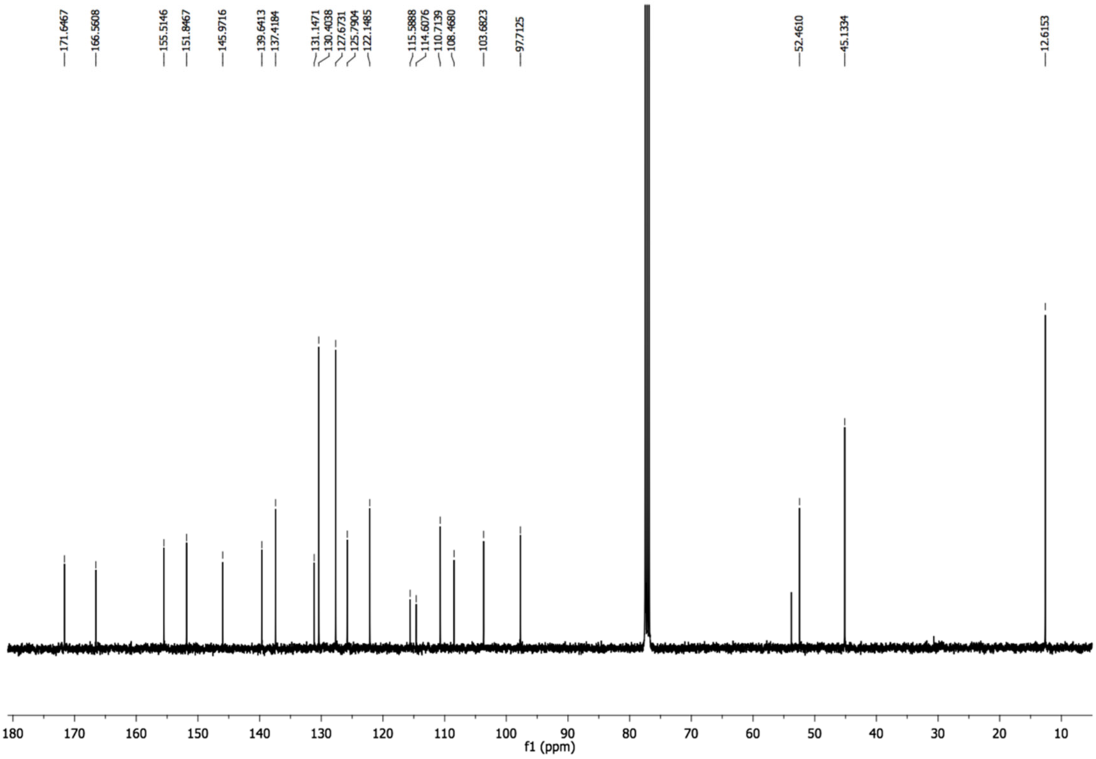

*^{13}C-NMR spectrum of **4** (CDCl₃, 100 MHz)*

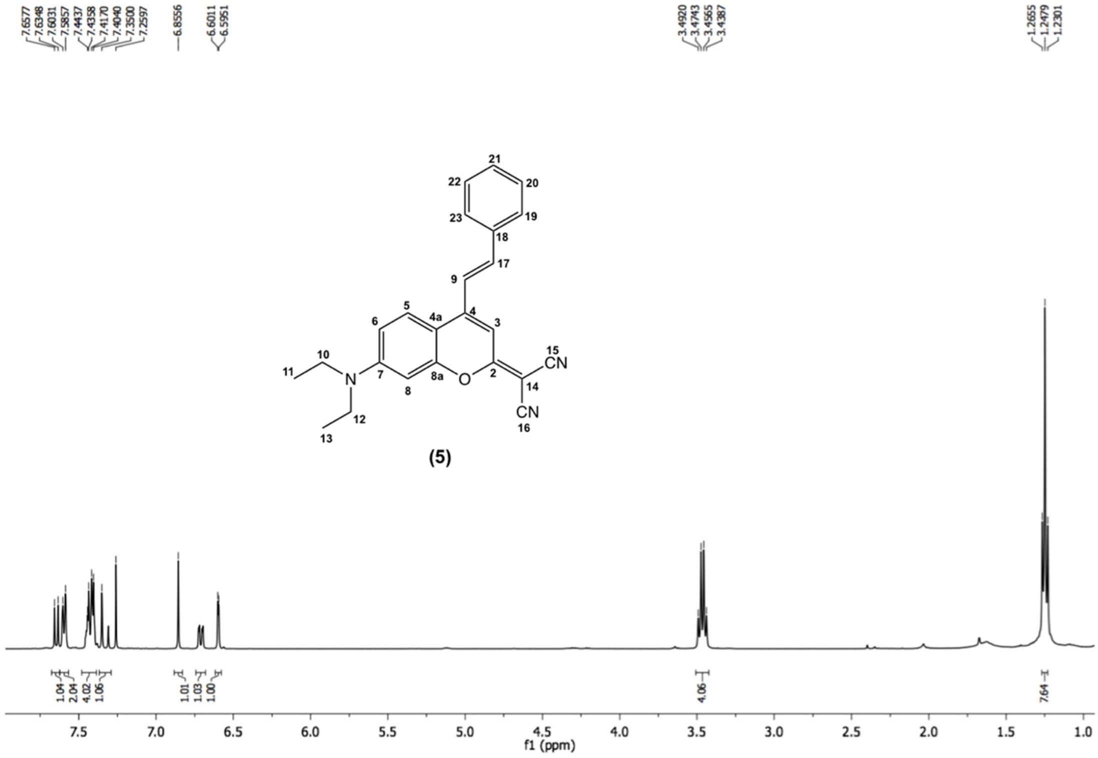

*^{1}H-NMR spectrum of **5** (CDCl3, 400 MHz)*

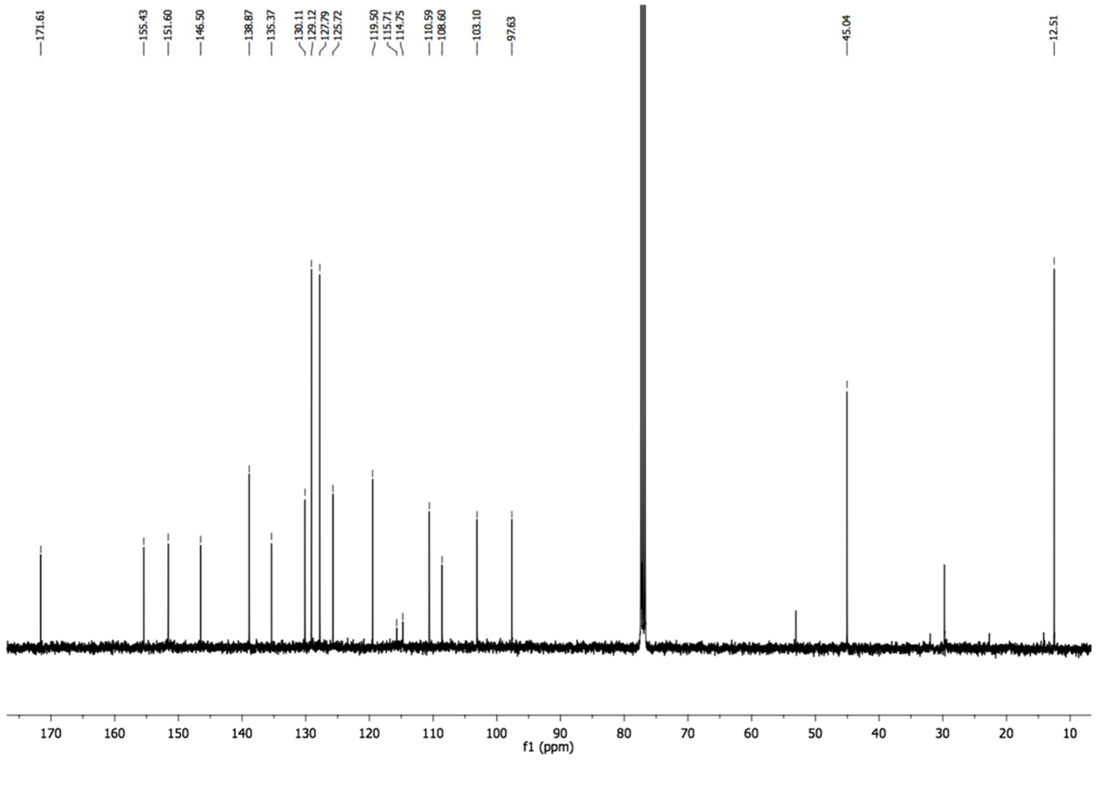

*^{13}C-NMR spectrum of **5** (CDCl3, 100 MHz)*

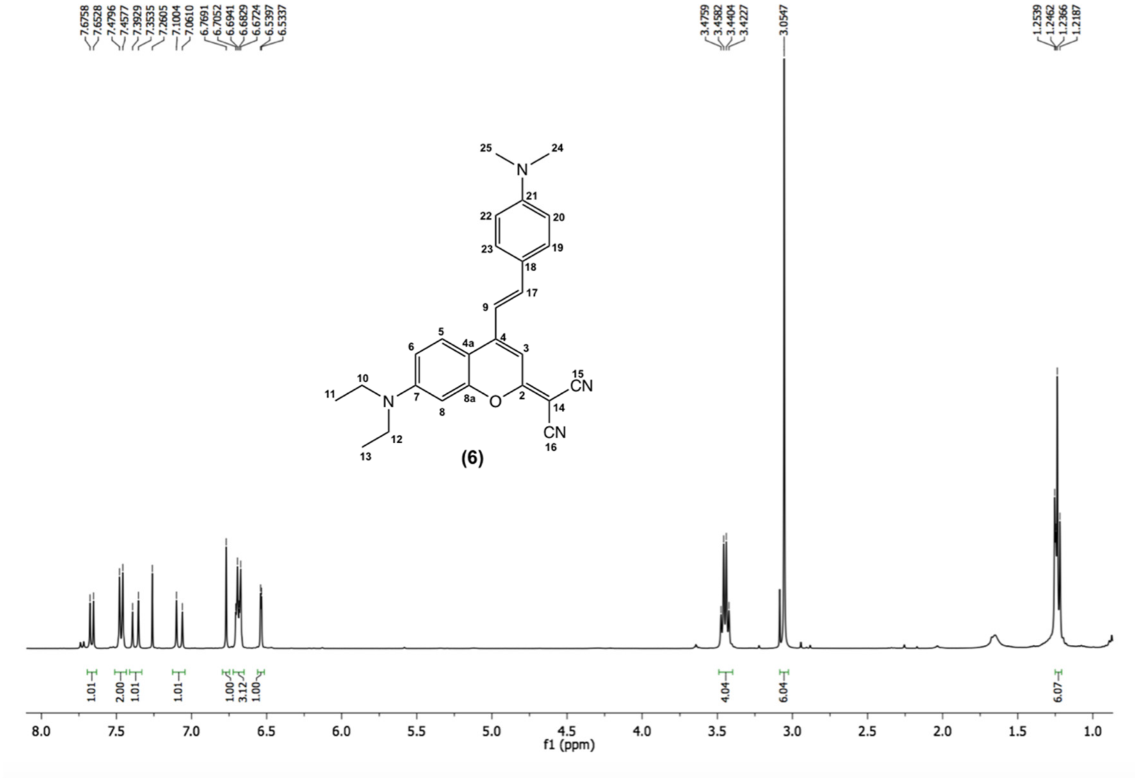

*^{1}H-NMR spectrum of **6** ($CDCl_3$, 400 MHz)*

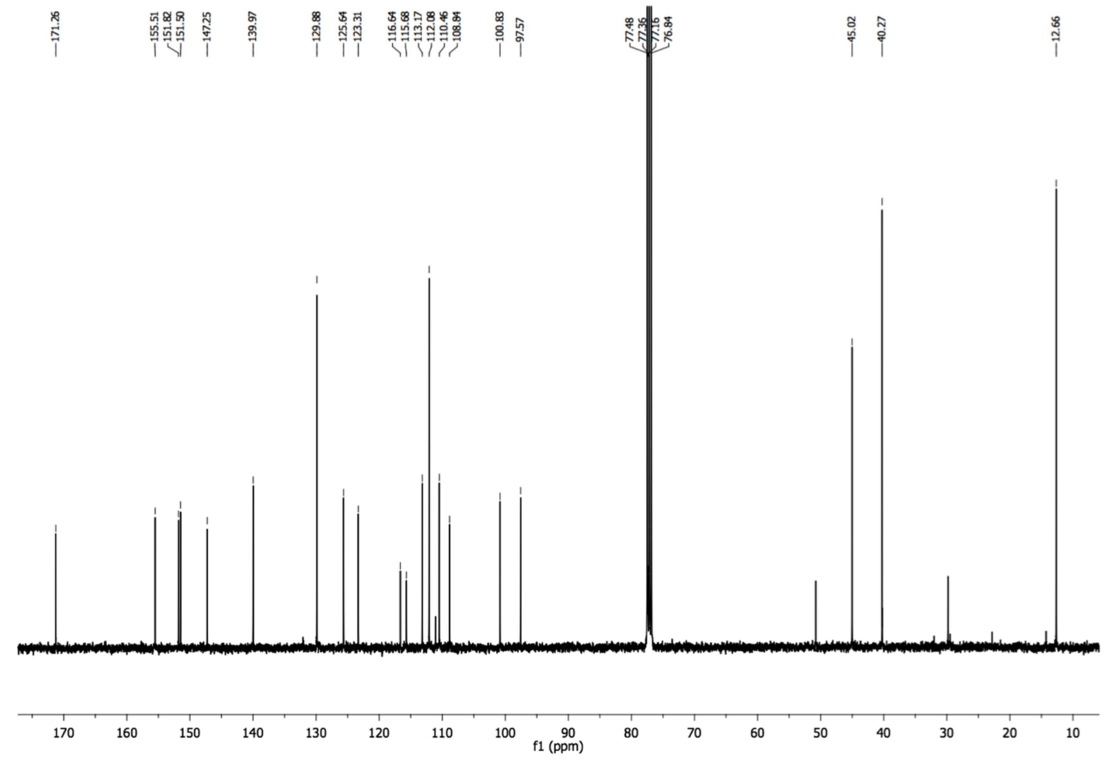

*^{13}C-NMR spectrum of **6** ($CDCl_3$, 100 MHz)*

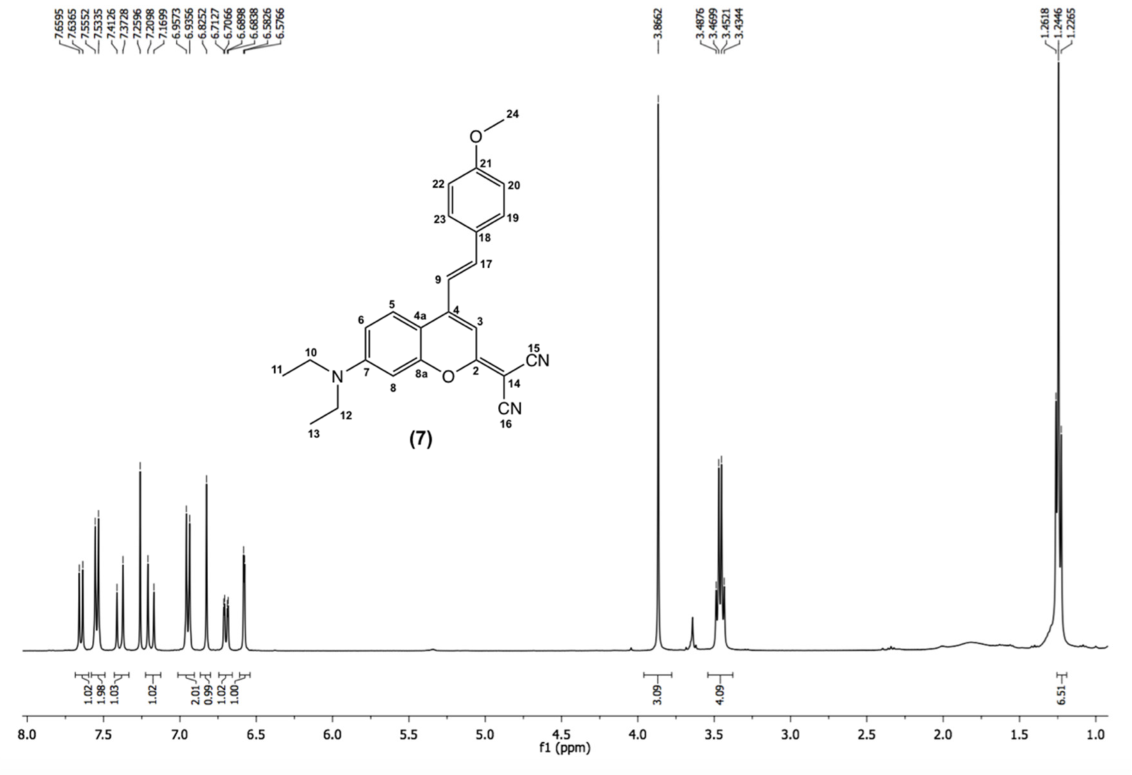

*^{1}H-NMR spectrum of **7** (CDCl₃, 400 MHz)*

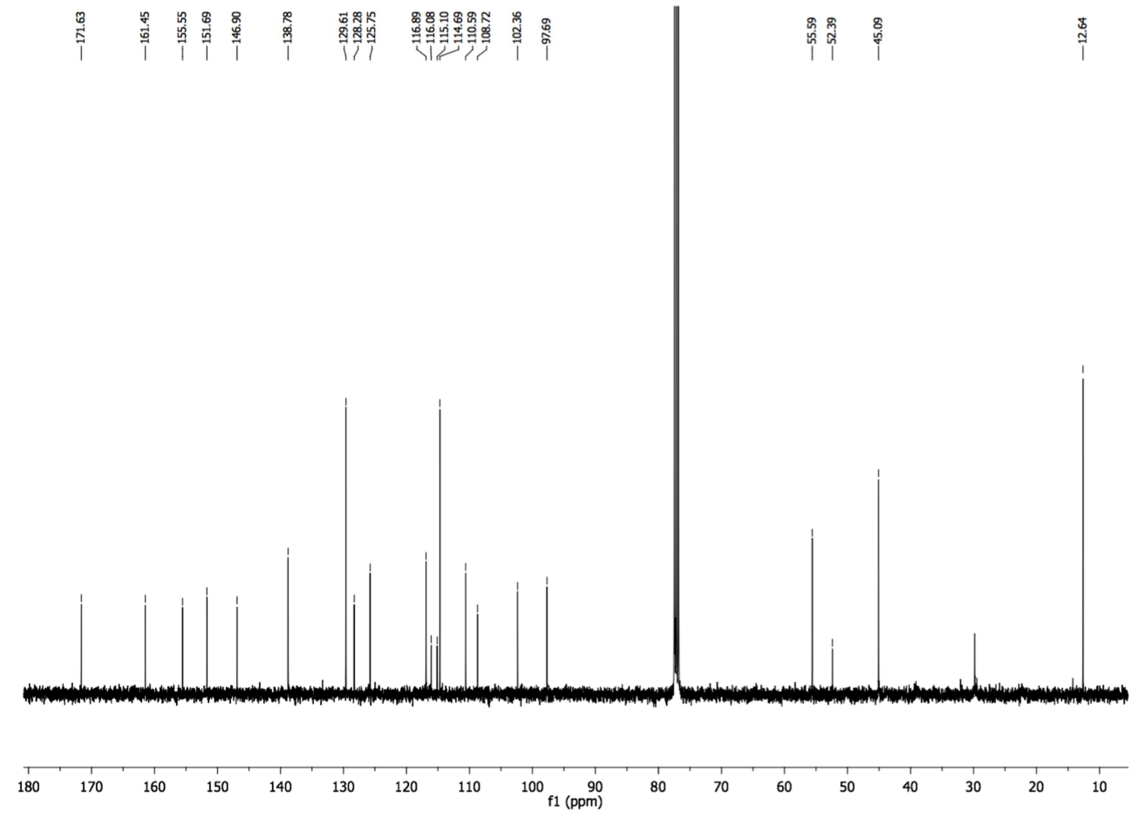

*^{13}C-NMR spectrum of **7** (CDCl₃, 100 MHz)*

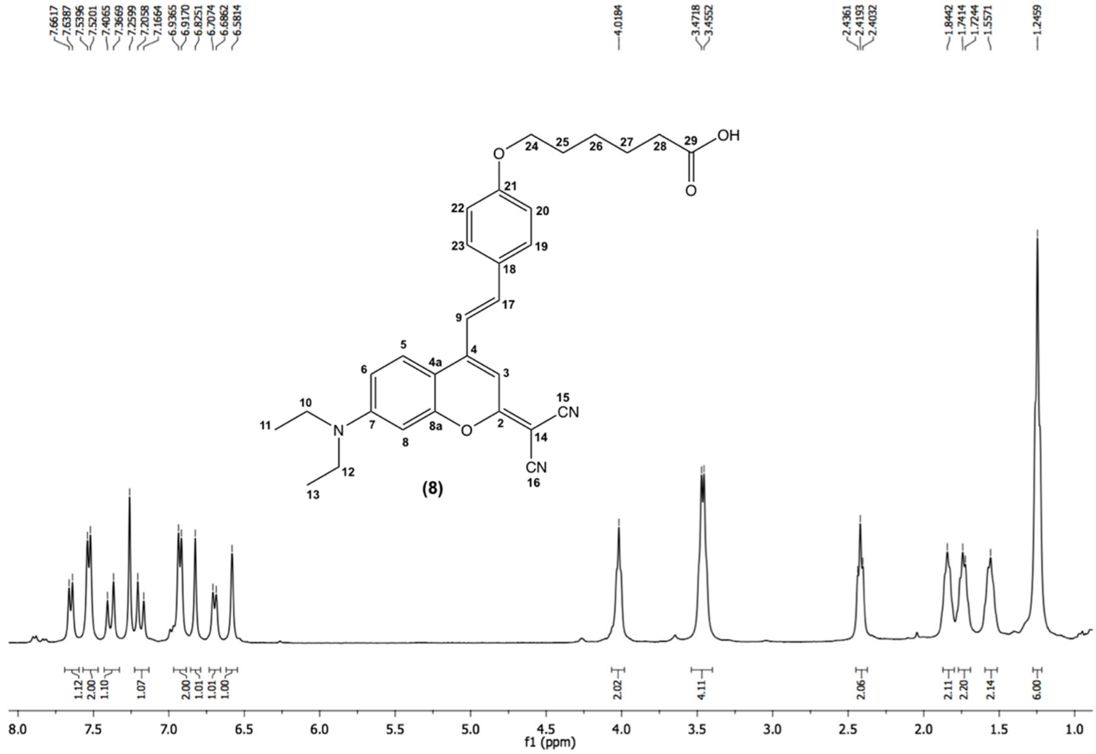

*^{1}H-NMR spectrum of **8** (CDCl₃, 400 MHz)*

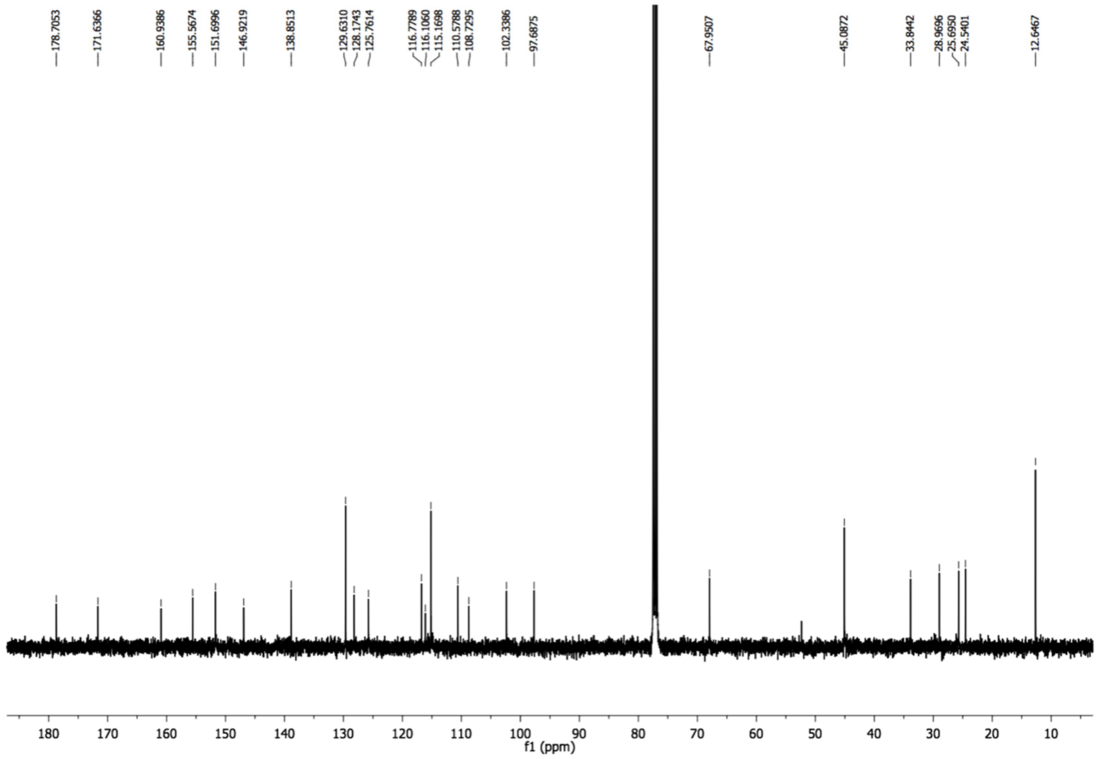

*^{13}C-NMR spectrum of **8** (CDCl₃, 100 MHz)*

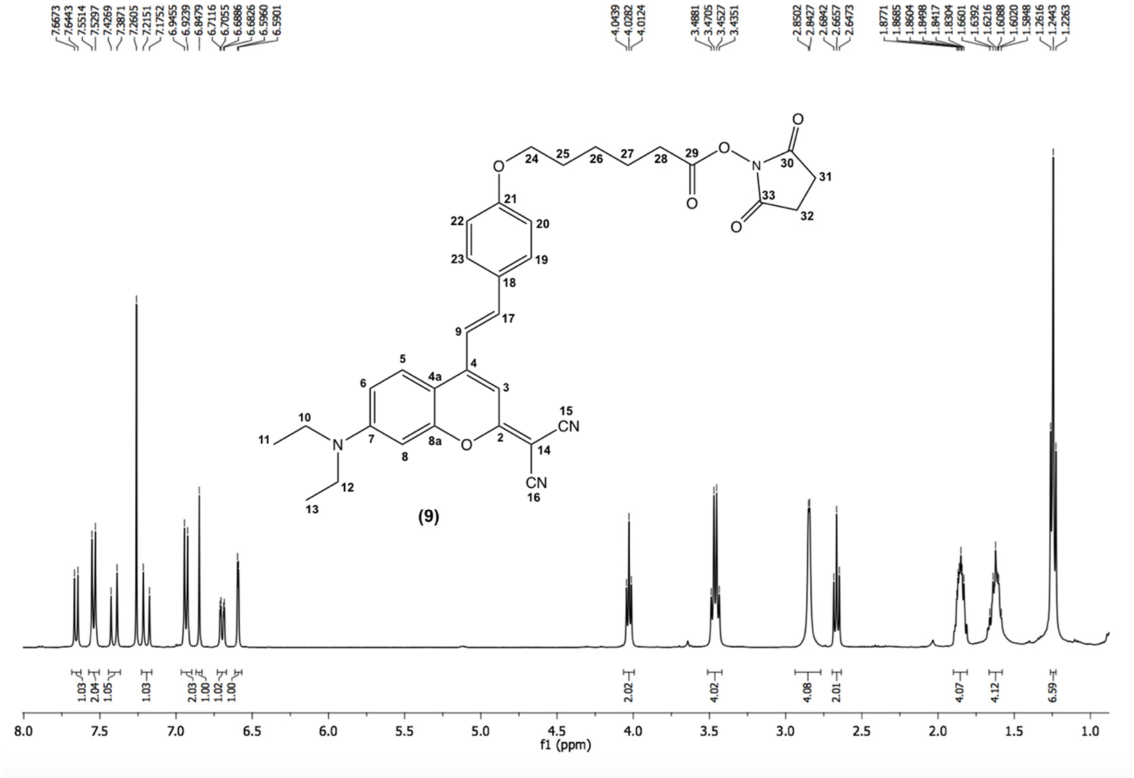

*^{1}H-NMR spectrum of **9** (CDCl₃, 400 MHz)*

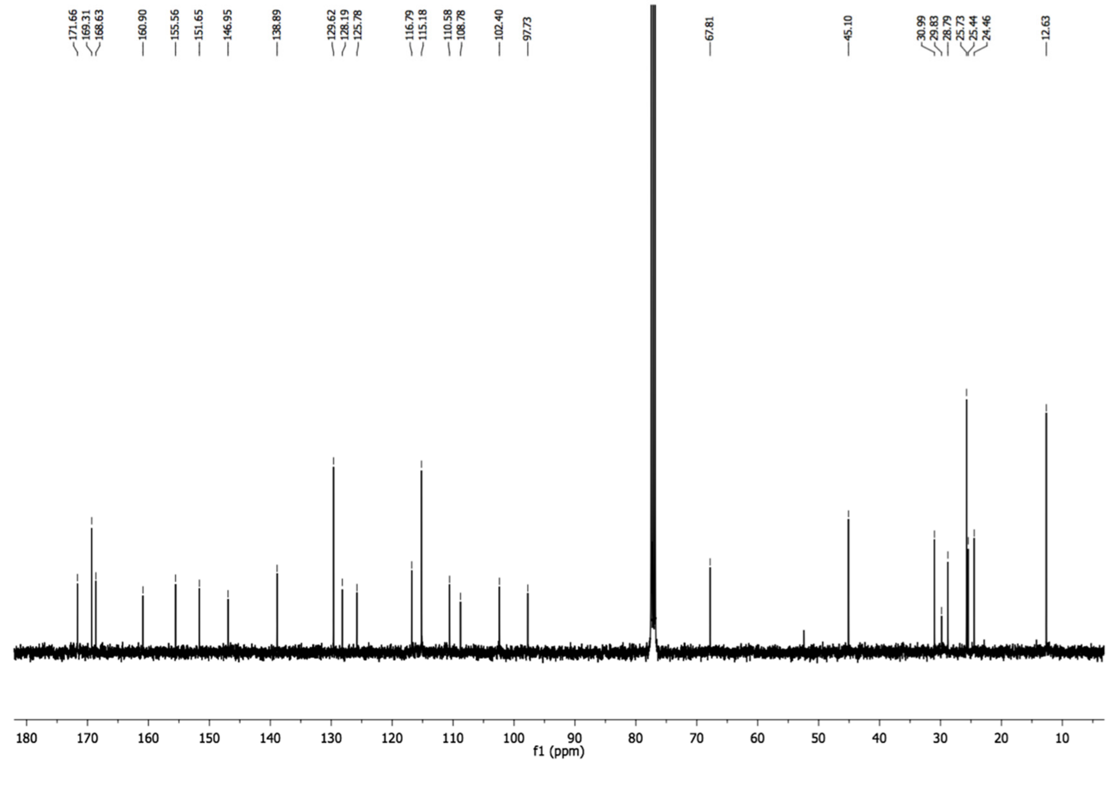

*^{13}C-NMR spectrum of **9** ($CDCl_3$, 100 MHz)*

2. Mass spectra

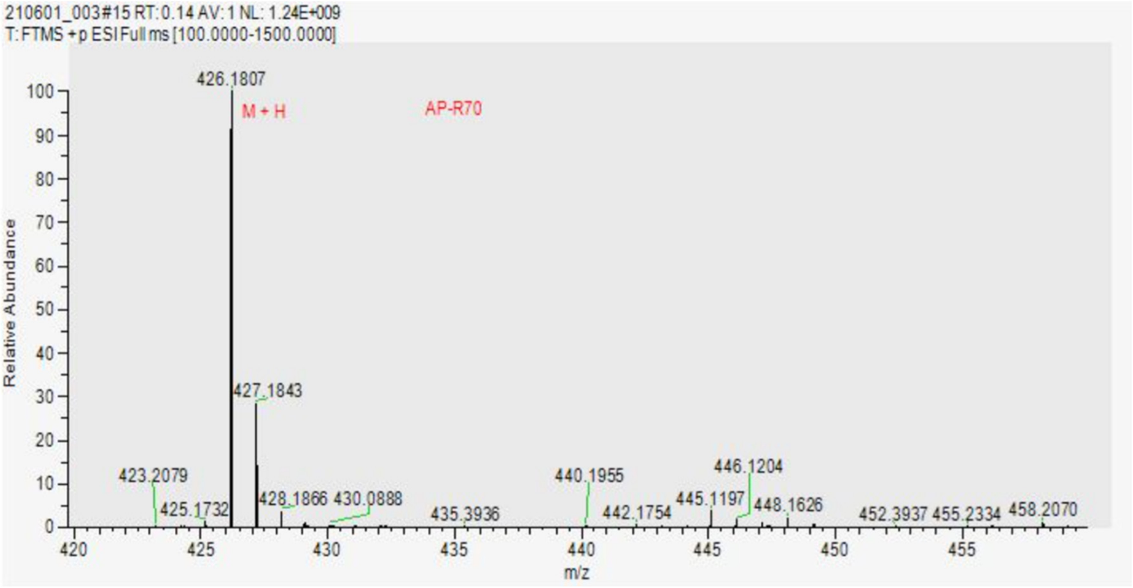

Mass spectrum of ***4***

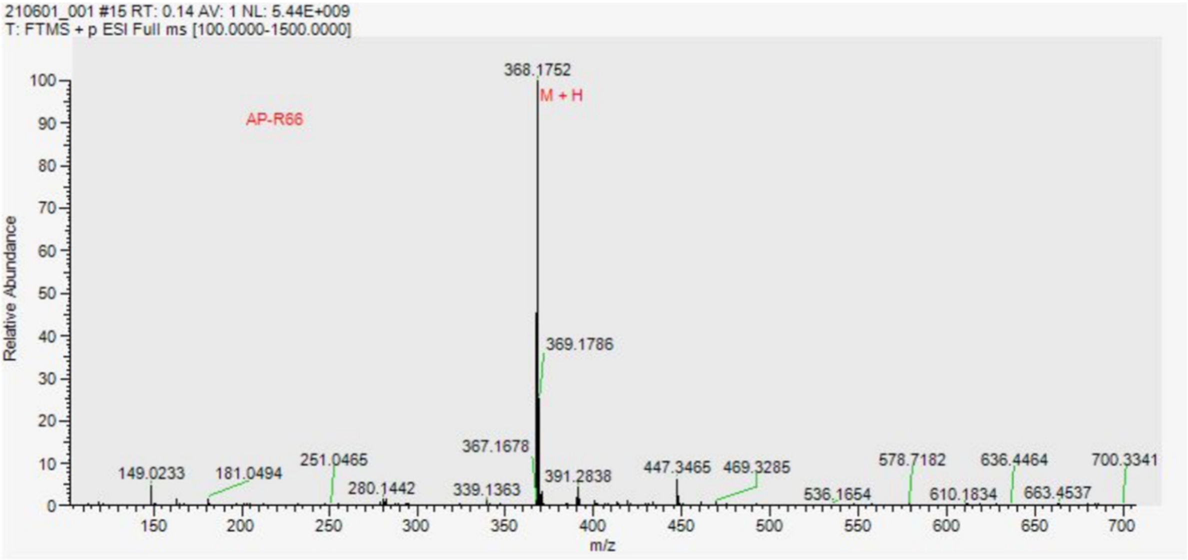

Mass spectrum of ***5***

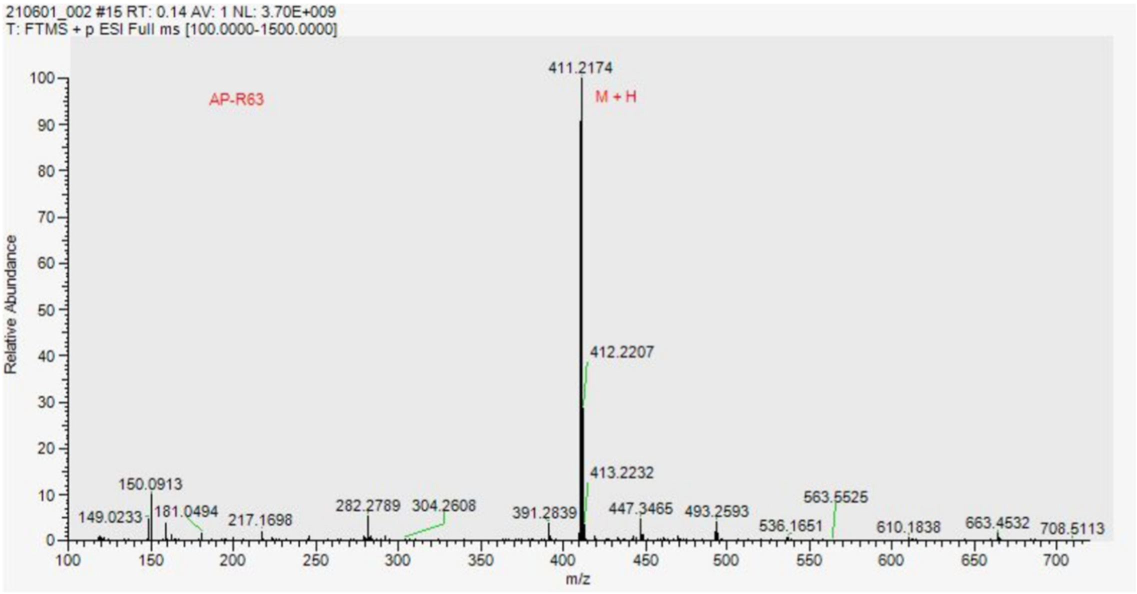

Mass spectrum of ***6***

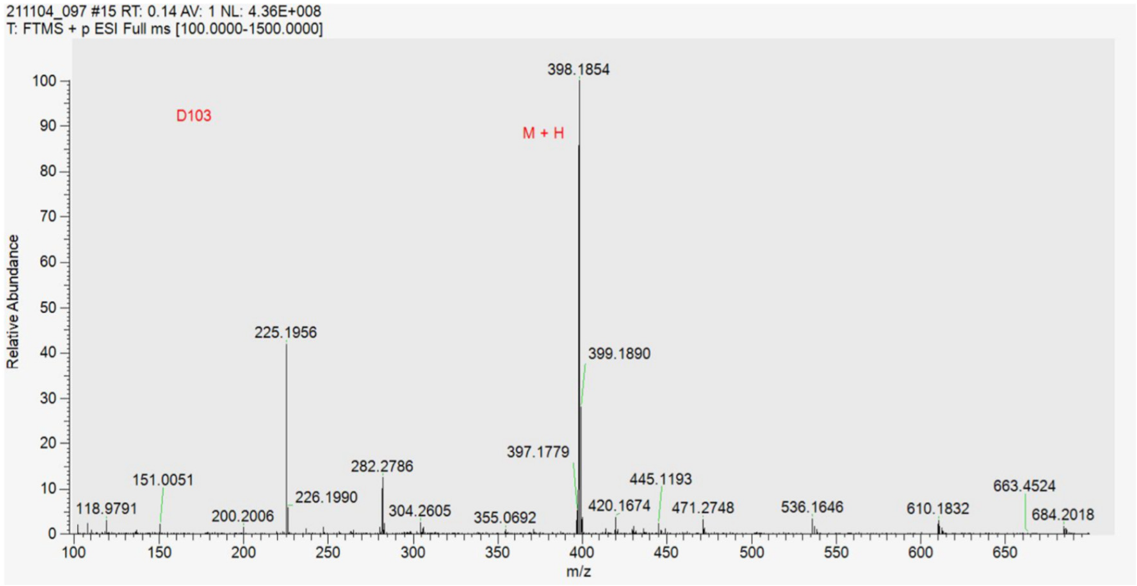

Mass spectrum of ***7***

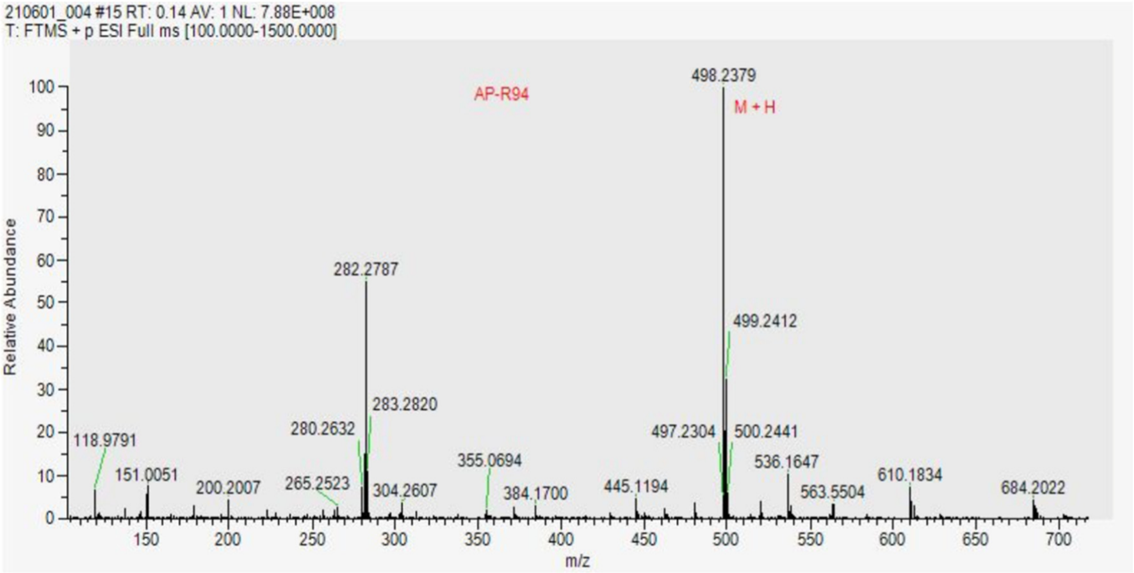

*Mass spectrum of **8***

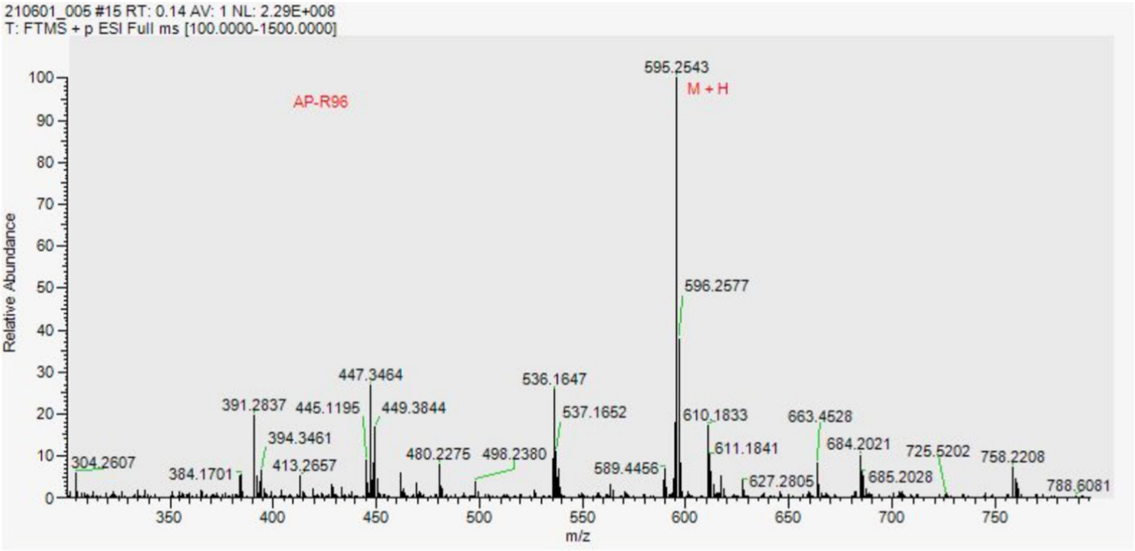

*Mass spectrum of **9***

3. Quantum chemical calculations

Quantum calculations were performed with the Gaussian 16 software package [44]. The hybrid PBE0 functional [45] with the standard 6-31G(d,p) basis set was used for geometry optimizations, both in the ground state and in the first singlet excited state, while the larger 6-311+G(d,p) basis set was employed for the spectra calculations. The PBE0 functional, besides being widely used for calculating ground state geometries, proved also to be very effective for TD-DFT calculation of excited-state structures [46]. In all cases solvent effects were taken into account by means of the implicit polarized continuum model (PCM) [47,48] No symmetry constraints are used during geometry optimizations. The frequency analysis confirmed the optimized structures as minima presenting all real valued frequencies.

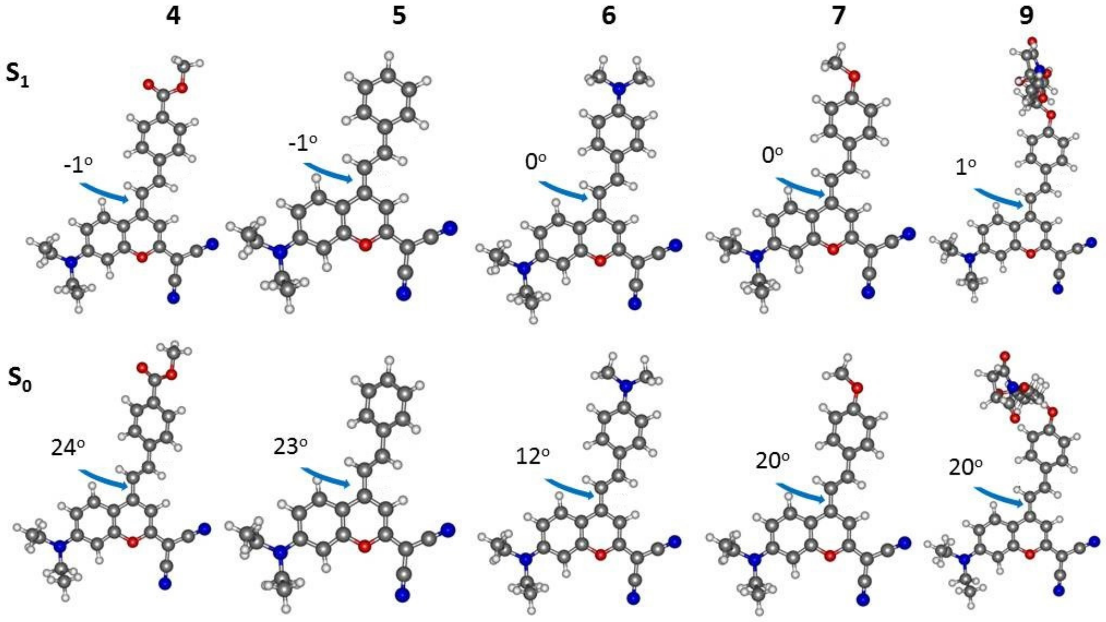

Figure S1. Optimized structures of the compounds in the ground state S_0 and in the first excited state S_1.

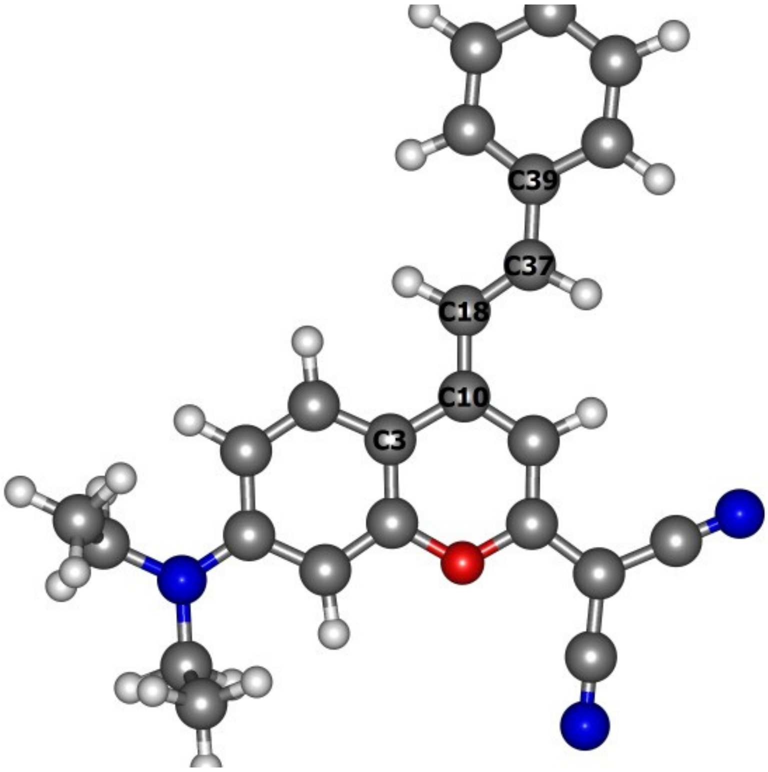

Figure S2. Numbering scheme adopted, common to all compounds.

Table S1. Selected bond distances (Å) and dihedral angle (°) of the compounds in the ground S_0 and excited S_1 states.

	r[C10-C18]	r[C18-C37]	r[C37-C39]	BLA	D_h[C3-C10-C18-C37]
S_0					
**4**	1.456	1.348	1.459	0.11	24.5
**5**	1.454	1.349	1.458	0.11	23.0
**6**	1.442	1.359	1.440	0.08	11.9
**7**	1.450	1.353	1.451	0.10	19.8
**9**	1.450	1.353	1.451	0.10	19.6
S_1					
**4**	1.409	1.382	1.432	0.04	-0.9
**5**	1.409	1.380	1.440	0.04	-0.6
**6**	1.417	1.385	1.430	0.04	-0.3
**7**	1.406	1.383	1.436	0.04	-0.5
**9**	1.405	1.383	1.436	0.04	0.7

Table S2. Calculated absorption data for the compounds and the main orbitals involved in the transitions

Compound 4

State	λ (nm)	*f*	Major MO → MO transitions
S_1	534.7	0.542	HOMO->LUMO (99%)
S_2	417.4	1.203	H-1->LUMO (98%)
S_3	355.4	0.302	H-2->LUMO (95%)
S_4	344.8	0.314	HOMO->L+1 (97%)
S_5	303.9	0.037	H-3->LUMO (93%)
S_6	300.1	0.065	H-4->LUMO (71%), HOMO->L+2 (21%)
S_7	289.6	0.231	H-1->L+1 (41%), HOMO->L+2 (42%)
S_8	280.3	0.028	H-4->LUMO (17%), H-1->L+1 (53%), HOMO->L+2 (22%)
S_9	270.7	0.001	H-1->L+5 (17%), HOMO->L+5 (77%)
S_{10}	270.6	0.000	H-5->LUMO (78%), H-5->L+1 (17%)

Compound 5

State	λ (nm)	*f*	Major MO → MO transitions
S_1	513.3	0.618	HOMO->LUMO (99%)
S_2	415.0	1.140	H-1->LUMO (98%)
S_3	349.3	0.164	H-2->LUMO (96%)
S_4	316.2	0.244	HOMO->L+1 (94%)
S_5	304.1	0.023	H-3->LUMO (95%)
S_6	292.8	0.062	H-4->LUMO (76%), HOMO->L+2 (17%)
S_7	278.8	0.347	H-4->LUMO (14%), H-1->L+1 (15%), HOMO->L+2 (61%)
S_8	271.8	0.002	H-1->L+5 (15%), HOMO->L+5 (76%)
S_9	267.3	0.045	H-1->L+1 (65%), HOMO->L+2 (15%)
S_{10}	262.2	0.083	H-1->L+1 (16%), HOMO->L+3 (10%), HOMO->L+4 (64%)

Compound 6

State	λ (nm)	*f*	Major MO → MO transitions
S_1	543.9	1.442	HOMO->LUMO (98%)
S_2	495.6	0.712	H-1->LUMO (99%)
S_3	360.1	0.017	H-2->LUMO (95%)
S_4	317.1	0.130	H-3->LUMO (24%), HOMO->L+1 (65%)
S_5	310.8	0.054	H-3->LUMO (39%), H-1->L+1 (25%), HOMO->L+1 (23%)
S_6	306.1	0.242	H-3->LUMO (31%), H-1->L+1 (63%)
S_7	299.1	0.022	H-4->LUMO (65%), HOMO->L+3 (20%)
S_8	284.6	0.106	H-4->LUMO (26%), HOMO->L+2 (40%), HOMO->L+3 (29%)
S_9	280.6	0.272	H-1->L+2 (13%), HOMO->L+2 (42%), HOMO->L+3 (31%)
S_{10}	278.7	0.003	H-2->L+5 (13%), H-1->L+5 (42%), HOMO->L+5 (38%)

Compound 7

State	λ (nm)	*f*	Major MO → MO transitions
S_1	507.2	0.634	HOMO->LUMO (99%)
S_2	452.3	1.324	H-1->LUMO (99%)
S_3	354.3	0.034	H-2->LUMO (96%)
S_4	312.7	0.209	HOMO->L+1 (88%)
S_5	297.9	0.049	H-3->LUMO (83%)
S_6	293.6	0.031	H-4->LUMO (78%)
S_7	283.6	0.212	H-4->LUMO (10%), H-1->L+1 (70%), HOMO->L+2 (15%)
S_8	275.0	0.179	H-1->L+1 (17%), HOMO->L+2 (66%)
S_9	273.6	0.005	H-2->L+5 (11%), HOMO->L+5 (75%)
S_{10}	263.5	0.078	H-1->L+2 (13%), HOMO->L+3 (31%), HOMO->L+4 (43%)

Compound 9

State	λ (nm)	*f*	Major MO → MO transitions
S_1	506.8	0.639	HOMO->LUMO (99%)
S_2	455.2	1.346	H-1->LUMO (99%)
S_3	354.5	0.031	H-2->LUMO (96%)
S_4	312.5	0.206	HOMO->L+1 (88%)
S_5	298.1	0.052	H-3->LUMO (80%)
S_6	294.4	0.026	H-4->LUMO (77%)
S_7	284.5	0.208	H-1->L+1 (73%), HOMO->L+3 (12%)
S_8	275.1	0.198	H-1->L+1 (14%), HOMO->L+3 (68%)
S_9	273.8	0.005	H-2->L+7 (11%), HOMO->L+7 (76%)
S_{10}	266.7	0.000	HOMO->L+2 (100%)

Table S3. Calculated emission data for the studied compounds and the main orbitals involved in the $S_1 \rightarrow S_0$ transitions.

Compound	λ (nm)	*f*	Major MO → MO transitions
4	630.2	0.438	HOMO->LUMO (99%)
5	590.0	0.505	HOMO->LUMO (99%)
6	583.0	1.512	HOMO->LUMO (99%)
7	577.1	0.539	HOMO->LUMO (99%)
9	576.2	0.546	HOMO->LUMO (99%)
